# Prognosis and management of new‐onset atrial fibrillation in critically ill patients

**DOI:** 10.1186/s12872-021-02039-w

**Published:** 2021-05-05

**Authors:** Jun Qian, Lijun Kuang, Fei Chen, Xuebo Liu, Lin Che

**Affiliations:** 1 Department of Cardiology, Shanghai Tongji Hospital, Tongji University School of Medicine, No.389, Xincun Rd, putuo District, Shanghai, 200065 China; 2 Department of Ultrasound, Luwan Branch, Ruijin Hospital, Shanghai Jiao Tong University School of Medicine, Shanghai, China

**Keywords:** Atrial fibrillation, Critical care, Mortality, Intensive care unit

## Abstract

**Introduction:**

The prognosis of new-onset atrial fibrillation (AF) compared with that of preexisting and non-AF remains controversial. The purpose of this study was to evaluate the effect of new-onset AF compared with preexisting and non-AF on hospital and 90-day mortality.

**Methods:**

A retrospective cohort study was performed using data obtained from the Medical Information Mart for Intensive Care III database. The primary outcome was 90-day mortality. Secondary outcomes included hospital mortality, hospital and intensive care unit (ICU) length of stay, and acute kidney injury. Logistic and Cox regression analyses were performed to evaluate the relationship between new-onset AF and study outcomes.

**Results:**

A total of 38,159 adult patients were included in the study. The incidence of new-onset AF was 9.4%. Ninety-day mortality, hospital mortality, and hospital and ICU length of stay in patients with new-onset and preexisting AF were significantly increased compared with those in patients with non-AF patients (all p < 0.001). After adjusting for patient characteristics, new-onset AF remained associated with increased 90-day mortality compared with non-AF (adjusted hazard ratio (HR) 1.37, 95% confidence interval (CI) 1.26 to 1.50; p < 0.01) and preexisting AF (adjusted HR 1.12; 95%-CI 1.02 to 1.23; p < 0.01). Patients in the surgical intensive care unit (SICU) had significantly higher 90-day mortality than patients in the coronary care unit (adjusted HR 1.30; 95% CI 1.31 to 1.51; p < 0.001).

**Conclusions:**

Critically ill patients with new-onset AF have significantly increased hospital and 90-day mortality compared with patients with preexisting and non-AF. Patients with new-onset AF in the ICU, especially those in the SICU, require robust management measures.

**Supplementary Information:**

The online version contains supplementary material available at 10.1186/s12872-021-02039-w.

## Background

Atrial fibrillation (AF) may present as irregular, rapid, electrical and mechanical activation of the atria, resulting in asynchronous contraction of the atria that may promote thromboembolism formation [1]. AF is the most common arrhythmia in clinical settings, especially in intensive care unit (ICU) patients [2]. AF is mainly divided into paroxysmal AF, persistent AF, long-term persistent AF, and permanent AF [3]. Preexisting AF is very common among ICU patients, while new-onset AF is also a frequent complication in the ICU with an incidence of approximately 5% [4–6].

Causes of new-onset AF might include electrolyte disturbances, fluid imbalances, neurohormonal disturbances, arrhythmic drug use, and inflammatory reactions [4]. Chronic heart failure, hypertension, valvular disease, and myocardial infarction trigger a variety of common inflammatory pathways, activation of the renin-angiotensin system and production of reactive oxygen species that lead to atrial fibrosis and further promote the occurrence of AF [7]. In patients who have undergone surgery and had a recent myocardial infarction, AF was often associated with poor outcomes, such as an increased risk of stroke [8–12]. However, evidence of the effects of new-onset AF, preexisting and non-AF with prognosis in ICU patients is limited and contradictory.

Preexisting and new-onset AF have been shown to be associated with all-cause mortality [13, 14]. However, several studies have shown that preexisting and new-onset AF may not be independently associated with hospital mortality [15, 16]. A cohort study including more than 1300 critically ill patients with persistent arrhythmias found that AF was not associated with increased mortality [17]. Many previous studies had limited sample sizes, and their conclusions were controversial. The aim of this study was to identify the associations of new-onset AF, preexisting AF, and non-AF with hospital and 90-day mortality among ICU. This retrospective observational study was performed in accordance with the STROBE reporting checklist.

## Methods

### Database access

We performed a retrospective study based on the ‘Medical Information Mart for Intensive Care (MIMIC) III’ database [18]. The database includes comprehensive clinical information of inpatients treated at the Beth Israel Deaconess Medical Center (BIDMC) in Boston, Massachusetts from June 1, 2001 to October 31, 2012. The use of this database was approved by the BIDMC Institutional Review Board. Our data extraction was performed by Dr. Qian who obtained access to the database (certificate code: 32,299,459). https://mimic.physionet.org/gettingstarted/access/.

### Patient and data extraction

**Fig. 1:**
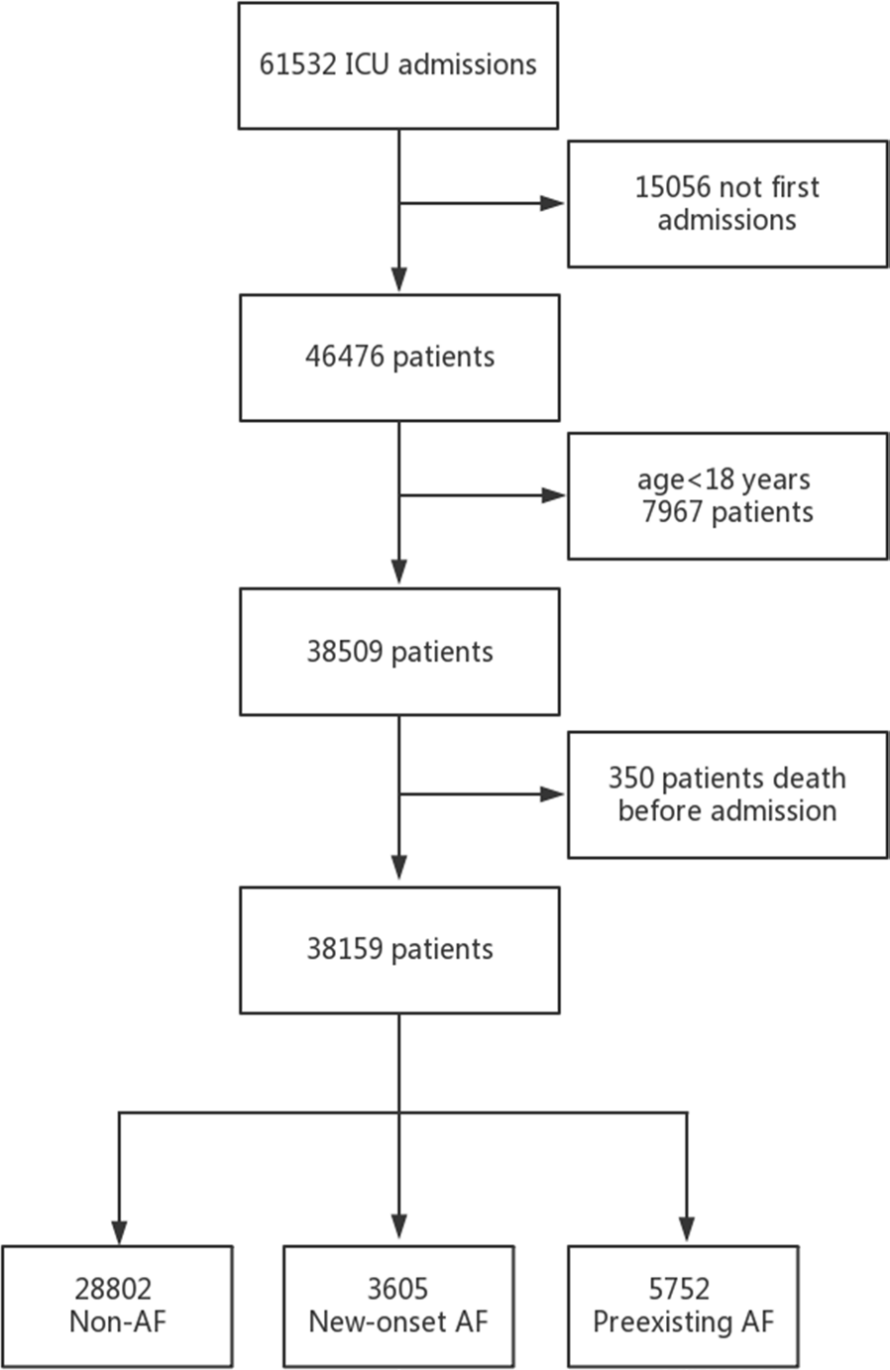
Flow chart of the study population. *ICU* intensive care unit, *AF* atrial fibrillation

The exclusion criteria were age younger than 18 years and death before hospitalization. All other patients (≥ 18 years) alive at admission were included in the study. For patients who were admitted multiple times, only the data from first ICU admission was retained. The detailed research flow chart is shown in Fig. 1. Information extracted from the database included age, gender, comorbidities on admission, type of ICU, laboratory tests performed on admission, Sequential Organ Failure Assessment (SOFA) score, medications during hospitalization and length of stay (LOS). The type of ICU each patient was admitted to was determined by the physician based on pathological state of each patient at the time of admission. All drugs referred to in our study were administered at the time of admission and continued after discharge.

### Definitions and outcomes

We divided all patients into three groups: non-AF, new-onset AF, and preexisting AF. New-onset AF was defined as the first diagnosis during hospitalization based on a 12-lead electrocardiogram. Patients with a diagnosis of AF before hospital admission were identified as preexisting AF. Patients with neither AF diagnosis were defined as non-AF. The primary outcome was 90-day mortality. Secondary outcomes included hospital mortality, hospital and ICU LOS, and acute kidney injury during hospitalization. Acute kidney injury was defined as a serum creatinine (Scr) level that was 1.5 times higher than baseline [19].

### Statistical analysis

Continuous variables are expressed as the mean ± standard deviation (SD) or median (interquartile range) as appropriate and were compared by analysis of variance or the Mann-Whitney *U* test. Categorical variables are presented as percentages and were, compared by the chi-square test. The univariate Kaplan-Meier method was used to estimate the 90-day mortality among the three groups. Ninety-day and hospital mortality were assessed using a Cox regression model and a logistic regression model. We used two different models to adjust for potential confounders: (1) model 1, which included age, gender, type of ICU, and comorbidities on admission including hypertension, diabetes, coronary heart diseases, congestive heart failure, hyperlipidemia, chronic obstructive pulmonary disease (COPD), cerebral infarction, pulmonary embolism, sepsis, and hypothyroidism and (2) model 2, which included the SOFA score, laboratory tests performed on admission including the white blood cell (WBC) count, hemoglobin (HB) level, Scr, and medications administered during hospitalization including β blockers, statins, amiodarone, non-dihydropyridine calcium channel blockers (CCB), digoxin, and warfarin in addition to the above mentioned variables from model 1. The significance level was set at 0.05, and all analyses were two-sided. Statistical analyses were performed using Statistical Package for the Social Sciences (SPSS) 22.0 (SPSS Inc., Armonk, NY, USA) and R software (version 3.6.1; http://www.R-project.org, R Foundation for Statistical Computing, Vienna, Austria).

## Results

### Patients characteristics

A total of 38,159 patients were enrolled in the study. The three groups of non-AF, new-onset AF, and preexisting AF included 28,802, 3605, and 5752 patients, respectively. The incidence of new-onset AF during hospitalization was 9.4%. Table 1 shows the baseline characteristics for the 3 groups. The age of patients with preexisting and new-onset AF was significantly older than that of patients with non-AF (p < 0.001). Patients with new-onset AF had an increased prevalence of hypertension, diabetes, coronary heart disease, and hyperlipidemia. The prevalence of new-onset AF was the highest in the cardiac surgical recovery unit (CSRU). Patients with new-onset AF had the highest use of amiodarone and statins, while those with preexisting AF had the highest use of β blockers, digoxin, and warfarin.

**Table 1:** Baseline characteristics of the study population

Variable	Non-AF (n = 28,802)	New-onset AF (n = 3605)	Preexisting AF (n = 5752)	p value
Age(years)	60.30 ± 17.63	72.96 ± 11.75	75.53 ± 11.38	< 0.001
Male(n(%))	16,249 (56.42)	2162 (59.97)	3186 (55.39)	< 0.001
*Comorbidities(n(%))*				
Hypertension	11,911 (41.35)	1929 (53.51)	2691 (46.78)	< 0.001
Diabetes	7109 (24.68)	1054 (29.32)	1648 (28.65)	< 0.001
CHD	7691 (26.70)	1857 (51.51)	2239 (38.92)	< 0.001
Congestive heart failure	5054 (17.55)	1258 (34.90)	2883 (50.12)	< 0.001
Hyperlipidemia	4399 (15.27)	894 (24.80)	1124 (19.54)	< 0.001
COPD	539 (1.87)	69 (1.91)	168 (2.92)	< 0.001
Previous cerebral infarction	1246 (4.33)	253 (7.02)	525 (9.13)	< 0.001
Pulmonary embolism	746 (2.59)	58 (1.61)	143 (2.49)	0.002
Sepsis	2371 (8.23)	279 (7.74)	701 (12.19)	< 0.001
Hypothyroidism	2477 (8.60)	407 (11.29)	686 (11.93)	< 0.001
*Type of ICU (n(%))*				< 0.001
CCU	3918 (13.60)	545 (15.12)	1147 (19.94)	
CSRU	4682 (16.26)	1624 (45.05)	1269 (22.06)	
MICU	10,737 (37.28)	798 (22.14)	1990 (34.60)	
SICU	5059 (17.56)	390 (10.82)	846 (14.71)	
TSICU	4406 (15.30)	248 (6.88)	500 (8.70)	
*Initial laboratory data*				
WBC, 10^3^/uL	11.74 ± 5.80	12.26 ± 5.68	12.07 ± 5.94	< 0.001
Hemoglobin, mg/dl	11.06 ± 1.99	10.44 ± 1.90	10.70 ± 1.92	< 0.001
Creatinine, mg/dl	1.2 (0.7–1.4)	1.2 (0.8–1.4)	1.4 (0.8–1.6)	< 0.001
SOFA score	3.7 (2–6)	4.6 (2–6)	4.2 (2–6)	< 0.001
*Rhythm or rate control drugs(n(%))*				
β-blockers	13,638 (47.35)	2661 (73.81)	4371 (75.99)	< 0.001
Amiodarone	882 (3.06)	1455 (40.36)	1807 (31.42)	< 0.001
Nondihydropyridine CCB	978 (3.40)	423 (11.73)	1560 (27.12)	< 0.001
Digoxin	318 (1.10)	243 (6.75)	926 (16.10)	< 0.001
Statin (n(%))	5788 (20.10)	1459 (40.47)	1684 (29.28)	< 0.001
Warfarin (n(%))	2204 (7.65)	1206 (33.45)	2501 (43.48)	< 0.001

*AF* atrial fibrillation, *CHD* coronary heart disease, *COPD* chronic obstructive pulmonary disease, *CCU* coronary care unit, *CSRU* cardiac surgery recovery unit, *MICU* medical intensive care unit, *TSICU* trauma/surgical intensive care unit, *WBC* white blood cell, *SOFA* sequential organ failure assessment, *CCB* calcium channel blocker

### Clinical outcomes

**Fig. 2:**
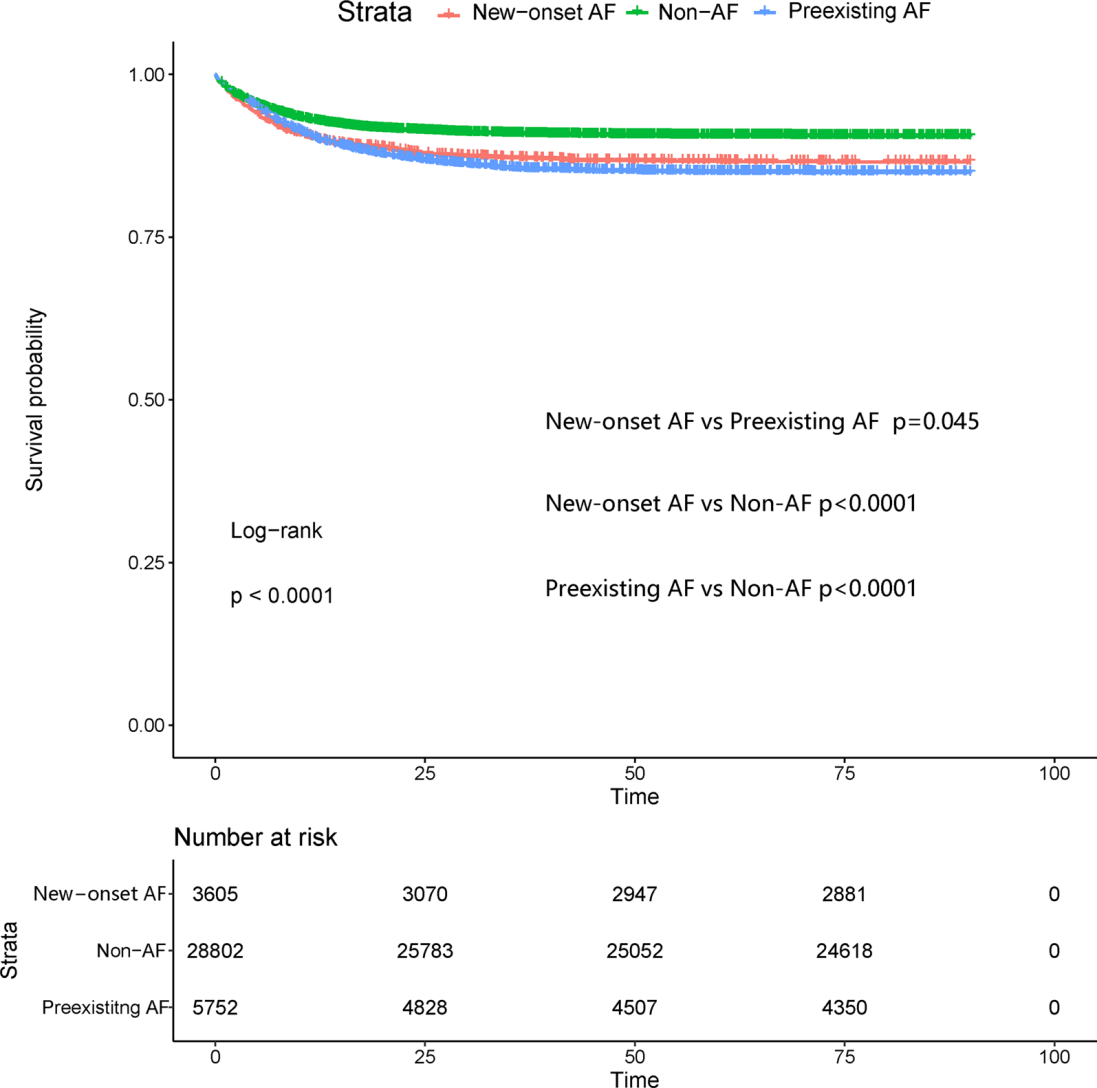
Kaplan-Meier survival curve for the primary outcome of 90-day mortality in the 3 study groups. *AF* atrial fibrillation. *Notes*: Log-rank test p < 0.0001

Table 2 shows the unadjusted outcomes among the 3 groups. The 90-day mortality, hospital mortality, hospital and ICU LOS of patients with new-onset and preexisting AF were significantly increased compared with those of patients with non-AF (all p < 0.001). Patients with non-AF had the highest proportion of acute kidney injury. The primary outcome of 90-day mortality among patients with non-AF, new-onset AF, and preexisting AF was 15.26%, 20.83 and 25.35%, respectively. In patients with non-AF, new-onset AF, and preexisting AF the hospital mortality rate was 9.52%, 13.34 and 14.97%, respectively. Figure 2 shows the Kaplan-Meier curve for survival probability in the 3 groups, which is consistent with Table 2. However, as presented in Table 3, the results of the multivariate Cox regression analyses show that new-onset AF was associated with an increased risk for 90-day mortality compared with non-AF and preexisting AF after adjustment using the two different models (model 2: compared with non-AF, adjusted hazard ratio (HR) 1.37, 95% confidence interval (CI) 1.26–1.50, p < 0.001; compared with preexisting AF, HR 1.12, 95 %CI 1.02–1.23, p = 0.019; model 1 is presented in Table 3). After adjusting two models using logistic regression analyses, new-onset AF was also associated with an increased risk of hospital mortality compared with non-AF and preexisting AF, as shown Table 4 (model 2: compared with non-AF, HR 1.61, 95 %CI 1.41–1.85, p < 0.001; compared with preexisting AF, HR 1.17, 95 %CI 1.01–1.35, p = 0.034).

**Table 2:** Unadjusted outcomes of the study population

	Non-AF (n = 28,802)	New-onset AF (n = 3605)	Preexisting AF (n = 5752)	p value
90-day mortality(n(%))	4394 (15.26)	751 (20.83)	1458 (25.35)	< 0.001
Hospital mortality(n(%))	2742 (9.52)	481 (13.34)	861 (14.97)	< 0.001
Hospital LOS(days)mean(IQR)	6.4 (3.8–11.2)	7.7 (5.1–12.0)	9 (5.6–15.0)	< 0.001
ICU LOS(days)mean(IQR)	2.0 (1.2–3.8)	2.3 (1.3–4.8)	2.9 (1.5–5.8)	< 0.001
AKI(n(%))	11,036 (38.3)	1238 (34.3)	1803 (31.3)	< 0.001

*AF* atrial fibrillation, *LOS* length of stay, *IQR* interquartile range, *ICU* intensive care unit, *AKI* acute kidney injury

**Table 3:** Adjusted hazard ratio of 90-day mortality comparing new-onset AF, non-AF and preexisting AF

Outcomes	Group	Hazard ratio	95% CI	p value
*90-days mortality*				
Crude	Non-AF	Ref		
	New-onset AF	1.41	1.30–1.52	< 0.001
Model 1	Non-AF	Ref		
	New-onset AF	1.33	1.22–1.44	< 0.001
Model 2	Non-AF	Ref		
	New-onset AF	1.37	1.26–1.50	< 0.001
Crude	Pre-existing AF	Ref		
	New-onset AF	0.81	0.75–0.89	< 0.001
Model 1	Pre-existing AF	Ref		
	New-onset AF	1.21	1.11–1.33	< 0.001
Model 2	Pre-existing AF	Ref		
	New-onset AF	1.12	1.02–1.23	0.019

Model 1 was adjusted by: age, gender, type of intensive care unit, hypertension, diabetes, coronary heart diseases, congestive heart failure, hyperlipidemia, chronic obstructive pulmonary disease (COPD), cerebral infarction, pulmonary embolism, sepsis, and hypothyroidism; Model 2 was adjusted by: except variables in model 1, the following have been added: Sequential Organ Failure Assessment(SOFA) score, laboratory tests performed on admission, including white blood cells (WBC), hemoglobin (HB), serum creatinine (Scr), and medications during hospitalization, including β blockers, statin, amiodarone, Non-dihydropyridine calcium channel blocker (CCB), digoxin, warfarin

*AF* atrial fibrillation, *Ref* reference

**Table 4:** Adjusted odds ratio of hospital mortality comparing new-onset AF, non-AF and preexisting AF

Outcomes	Group	Odds ratio	95% CI	p value
*Hospital mortality*				
Crude	Non-AF	Ref		
	New-onset AF	1.46	1.32–1.62	< 0.001
Model 1	Non-AF	Ref		
	New-onset AF	1.46	1.30–1.64	< 0.001
Model 2	Non-AF	Ref		
	New-onset AF	1.61	1.41–1.85	< 0.001
Crude	Preexisting AF	Ref		
	New-onset AF	0.88	0.77–0.99	0.029
Model 1	Preexisting AF	Ref		
	New-onset AF	1.31	1.15–1.49	< 0.001
Model 2	Preexisting AF	Ref		
	New-onset AF	1.17	1.01–1.35	0.034

Model 1 was adjusted by: age, gender, type of intensive care unit, hypertension, diabetes, coronary heart diseases, congestive heart failure, hyperlipidemia, chronic obstructive pulmonary disease (COPD), cerebral infarction, pulmonary embolism, sepsis, and hypothyroidism; Model 2 was adjusted by: except variables in model 1, the following have been added: Sequential Organ Failure Assessment(SOFA) score, laboratory tests performed on admission, including white blood cells (WBC), hemoglobin (HB), serum creatinine (Scr), and medications during hospitalization, including β blockers, statin, amiodarone, Non-dihydropyridine calcium channel blocker (CCB), digoxin, warfarin

*AF* atrial fibrillation, *Ref* reference

### Variables related to mortality

**Fig. 3:**
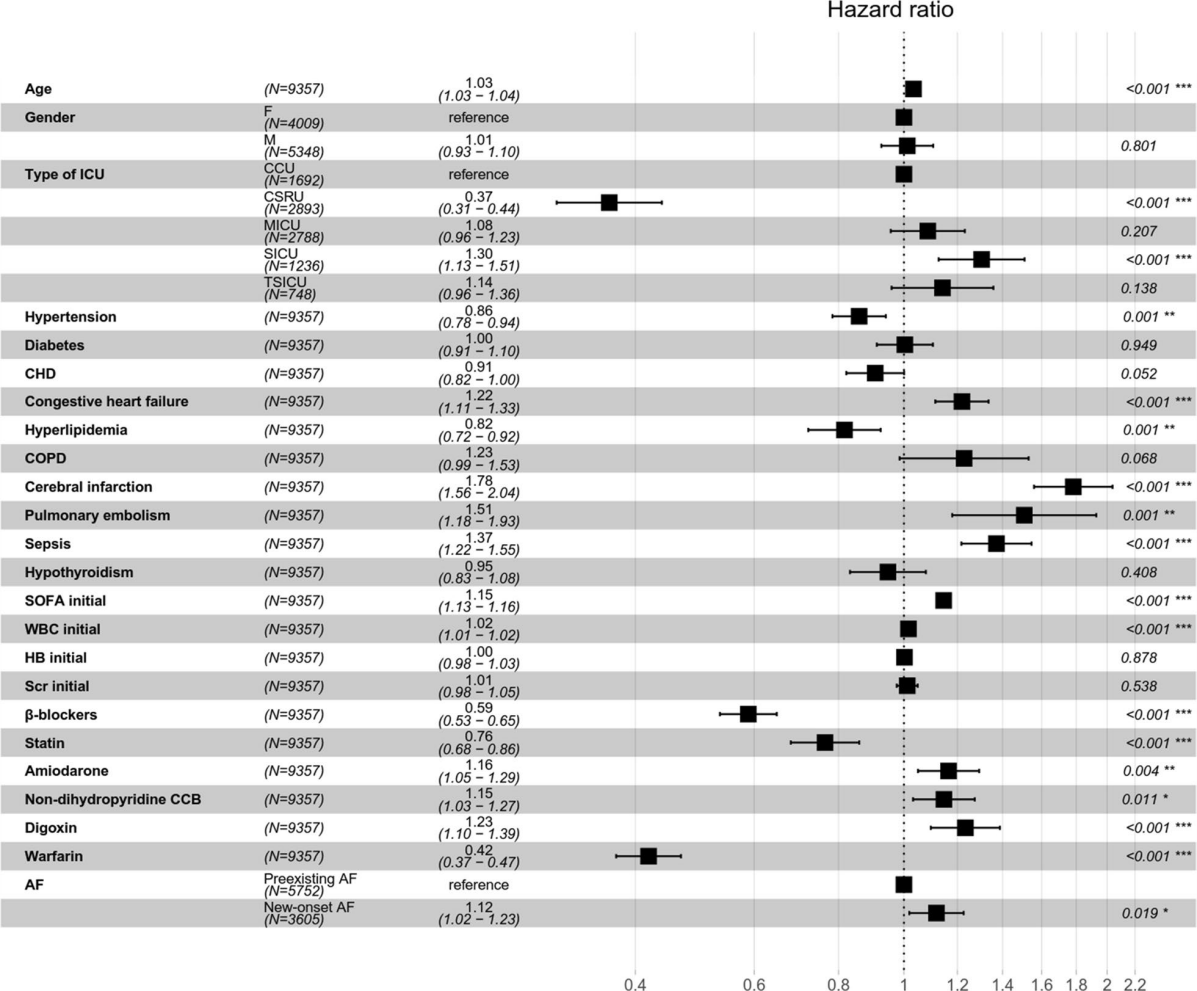
Multivariate Cox regression analyses in patients with AF for 90-day mortality after adjustment with model 2. *ICU* intensive care unit, *CHD* coronary heart disease, *COPD* chronic obstructive pulmonary disease, *SOFA* Sequential Organ Failure Assessment, *WBC* white blood cell, *HB* hemoglobin, *SCr* serum creatinine, *AF* atrial fibrillation

Figure 3 shows the associations between different variables and 90-day mortality among patients with AF. After adjusting for the variables in model 2, we found that age (HR 1.03, 95% CI 1.03–1.04, p < 0.001), congestive heart failure (HR 1.22, 95% CI 1.11–1.33, p < 0.001), cerebral infarction (HR 1.78, 95% CI 1.56–2.04, p < 0.001), pulmonary embolism (HR 1.51, 95% CI 1.18–1.93, p < 0.001), sepsis (HR 1.37, 95% CI 1.22–1.55, p < 0.001), SOFA score (HR 1.15, 95% CI 1.13–1.16, p < 0.001), WBC count (HR 1.02, 95% CI 1.01–1.02, p < 0.001), amiodarone (HR 1.16, 95% CI 1.05–1.29, p = 0.004), non-dihydropyridine CCB use (HR 1.15, 95% CI 1.03–1.27, p = 0.011), and digoxin (HR 1.23, 95% CI 1.10–1.39, p < 0.001) were associated with a significantly increased risk of 90-day mortality. In contrast, hypertension (HR 0.86, 95% CI 0.78–0.94, p = 0.001), hyperlipidemia (HR 0.82, 95% CI 0.72–0.92, p = 0.001), β-blockers use (HR 0.59, 95% CI 0.53–0.65, p < 0.001), statin use (HR 0.76, 95% CI 0.68–0.86, *p* < 0.001), and warfarin use (HR 0.42, 95% CI 0.37–0.47, p < 0.001) were protective factors for 90-day mortality in patients with AF. Patients with AF in the CSRU had a lower risk of 90-day mortality than patients with coronary care unit (CCU) (HR 0.37, 95% CI 0.31–0.44, p < 0.001), while patients with AF in the surgical intensive care unit (SICU) had an increased risk of 90-day mortality (HR 1.30, 95% CI 1.13–1.51, p < 0.001).

## Discussion

The incidence of new-onset AF is high in current critically ill patients and is associated with an increased mortality rate. However, strategies for treatment and management of these patients remain controversial. In this large retrospective study, we provide an important reference for the prognosis and management of new-onset AF. After adjusting for confounding factors in two models using Cox and logistic regression analyses, new-onset AF was found to be associated with significantly higher 90-day and hospital mortality than non-AF and preexisting AF. The prevalence of new-onset AF was approximately 9.4%. Furthermore, after adjusting for confounding factors in model 2 using multivariate Cox analyses, we also demonstrated that age, congestive heart failure, cerebral infarction, pulmonary embolism, sepsis, SOFA score, WBC count, amiodarone use, non-dihydropyridine CCB use, and digoxin use were associated with an increased risk of 90-day mortality in patients with AF, while hypertension, hyperlipidemia, β blockers use, statin use, warfarin use were protective factors for 90-day mortality in patients with AF. Patients with AF in the SICU had an increased risk of 90-day mortality compared with those in the CCU after adjusting for confounding variables.

Several previous studies have indicated that preexisting AF is associated with worse outcomes including higher rates of hospital and long-term mortality than non-AF patients [4, 9, 13]. In a previous retrospective study, preexisting AF was associated with an approximately 4 times increased risk of mortality compared with non-AF [20]. However, the effect of new-onset AF on mortality among critically ill patients remains controversial. Some prior studies did not support a significant correlation between new-onset AF and a high mortality risk [15, 21]. Another prospective cohort study showed that new-onset or preexisting AF was independently associated with increased mortality [14]. In our study, the univariate Kaplan-Meier method showed that the 90-day mortality rate among patients with preexisting AF was higher than that among those with new-onset and non-AF. However, consistent with previous research, after adjustment using two different models, new-onset AF was associated with higher 90-day mortality than preexisting and non-AF. This may be because multiple potential factors affect mortality associated with new-onset AF. Nevertheless, prior studies have failed to compare the mortality risk between patients with new-onset AF and those with preexisting AF [6, 22, 23]. The prevalence of new-onset AF in ICU patients in the current study is comparable to that reported in previous research [4, 6, 22, 24]. The reason why new-onset AF is associated with a poor prognosis is unclear. Infection and inflammation in critically ill patients may cause structural changes in the heart and accelerate electrical conduction [25, 26]. In addition, patients with electrolyte imbalances and those being treated with vasopressin therapy are also more likely to develop AF [27, 28]. An observational study revealed that the incidence of hemodynamic instability in patients with new-onset AF was significantly higher than that in patients with preexisting AF [24]. During AF, coordinated depolarization and contraction of the heart are disturbed by countless and disordered atrial electrical pulses, resulting in unstable contractions and loss of “atrial rhythmic beating”, thereby impairing cardiac output.

Notably, our study also demonstrated that age, congestive heart failure, cerebral infarction, pulmonary embolism, sepsis, SOFA score, WBC count, amiodarone use, non-dihydropyridine CCB use, and digoxin use were risk factors for 90-day mortality in critically ill patients with AF, while hypertension, hyperlipidemia, β blockers, statin and warfarin were protective factors with 90-day mortality (all p < 0.05). The incidence of previous cerebral infarction was significantly higher among patients with new-onset and preexisting AF than among patients with non-AF. Therefore, these patients may have had arrhythmic events prior to admission. However, we have no clear evidence to prove support this hypothesis. We focused on the effect of new-onset AF during hospitalization on 90-day prognosis. The final conclusion may be biased. Development of AF during a critically ill period is related to the presence of more serious disease before the onset of AF and to clinical deterioration after AF; therefore, it is difficult to identify a causal role of AF in affecting patient prognosis. However, comparison of our findings with those of other studies confirms that elderly age, congestive heart failure, cerebral infarction, pulmonary embolism, and sepsis were associated with an obviously increased mortality risk in critically ill patients with AF [27, 29]. After evaluating the data, we found that the SOFA score in more than 90% of patients was less than 10 points. However, the SOFA scores we included were those obtained on admission, and not all patients were in a very critical condition at admission. Condition of patients may undergo a series of changes during their hospital stays. The distribution of data is shown in the Additional file 1: Fig. 1. We believe that these data are still representative of patients with severe illness. The SOFA score was independently associated with 90-day mortality in patients with AF, which was also consistent with the founding of a previous study [30].

At present, there are still many controversies about the treatment strategy for patients with AF. Commonly used rate control drugs include non-dihydropyridine CCBs, β blockers and digoxin. Rhythm control drugs usually include magnesium and amiodarone, both of which have rhythm and rate control properties. In the current study, we found that β blockers, statins and warfarin were significantly associated with a decreased 90-day mortality risk, while amiodarone, non-dihydropyridine CCBs, and digoxin were associated with an increased risk. We evaluated the effect of warfarin on 90-day mortality in all patients with AF, including both those with new-onset and preexisting AF. Warfarin was associated with a decreased 90-day mortality risk in patients with new-onset and preexisting AF compared with those non-AF. β blockers have rate control, negative muscle strength and vasodilatory effects. They exert sympathetic effects by antagonizing β-1 receptors, resulting in decreased conductivity and a reduced effect of catecholamines on the myocardium [31]. In accordance with the present results, a previous study demonstrated that β blockers might be associated with lower hospital mortality in AF patients than amiodarone, non-dihydropyridine CCBs, and digoxin [32]. Digoxin slows the heart rate by increasing vagal nerve tension and may be related to hypotension. There is an association between digoxin and increased mortality, especially in patients with serum digoxin concentrations greater than 1.2ng/ml [33]. In addition, the vagal nerve effects of digoxin may be less effective in severe diseases characterized by a high catecholamine status [34]. Amiodarone inhibits adrenergic stimulation, blocks delayed current, and slows atrioventricular conduction. However, this drug still has many shortcomings, such as hypotension, pro-arrhythmic effects, and pulmonary toxicity. In this study, we found that the most widely used antiarrhythmic drugs in patients with AF were β blockers (75.2%), amiodarone (34.9%), CCBs (21.2%), and digoxin (12.5%). Among severely ill patients with AF in the United Kingdom, the most commonly used drug was amiodarone (> 80%), followed by β blockers (12%) [35]. Interestingly, in our study, statin use was also an independent protective factor for 90-day mortality in AF patients. This finding might be due to the anti-inflammatory effects of statins [36]. Patients with AF in SICU had increased 90-day mortality compared with those in the CCU after adjusting for confounding variables; thus, these patients may require more aggressive management.

### Limitations

There are several study limitations of this study. First, due to the retrospective nature of this study, a causal relationship between new-onset AF and mortality cannot be directly inferred. Such a conclusion requires further research to establish a definitive causal link. Second, all patients with new-onset AF were diagnosed based on a well-defined 12-lead electrocardiogram, which is the clearest evidence. However, we did not have access to hourly cardiac monitoring information, which inevitably could have led to missing a proportion of patients with new-onset AF, and to increasing the proportion of patients without AF. Nevertheless, an increased number of patients with non-AF would not significantly affect the results of this study. Third, although 2 different models were used to adjust for confounding factors, there may still be residual confounding factors that were not included. Finally, it is possible that health-care providers may choose pharmacological and electrical cardioversion during the onset of AF; this information was not available, which may have further increased the bias in this study.

## Conclusions

In conclusion, after adjusting for confounding factors using Cox and logistic regression analyses, critically ill patients with new-onset AF had a significantly increased risk of hospital and 90-day mortality compared with those with preexisting AF and non-AF. Patients with AF in the CSRU had a decreased risk of 90-day mortality, while patients with AF in the SICU had an increased risk of 90-day mortality, compared with those hospitalized in the CCU. Management of patients with new-onset AF patients in the ICU, especially in the SICU, requires robust measures. β blockers may be used as a first-line treatment for patients with AF in the ICU.

## Supplementary Information

**Additional file 1.** The distribution of SOFA score.

## Data Availability

The datasets used and analyzed during the current study are available from the corresponding author on reasonable request. The website of MIMIC database: https://mimic.physionet.org/.

## Abbreviations

AF: Atrial fibrillation
ICU: Intensive care unit
MIMIC: Medical Information Mart for Intensive Care
SOFA: Sequential organ failure assessment
LOS: Length of stay
COPD: Chronic obstructive pulmonary disease
WBC: White blood cells
HB: Hemoglobin
Scr: Serum creatinine
CCB: Calcium channel blocker
CSRU: Cardiac surgical recovery unit
HR: Hazard ratio
CI: Confidence interval
CCU: Coronary care unit
SICU: Surgical intensive care unit
