# Supplementary figures and images for: Prognosis and management of new‐onset atrial fibrillation in critically ill patients
Source: BMC Cardiovasc Disord. 2021 May 5;21:231. doi: 10.1186/s12872-021-02039-w (PMC8101157; doi:10.1186/s12872-021-02039-w)

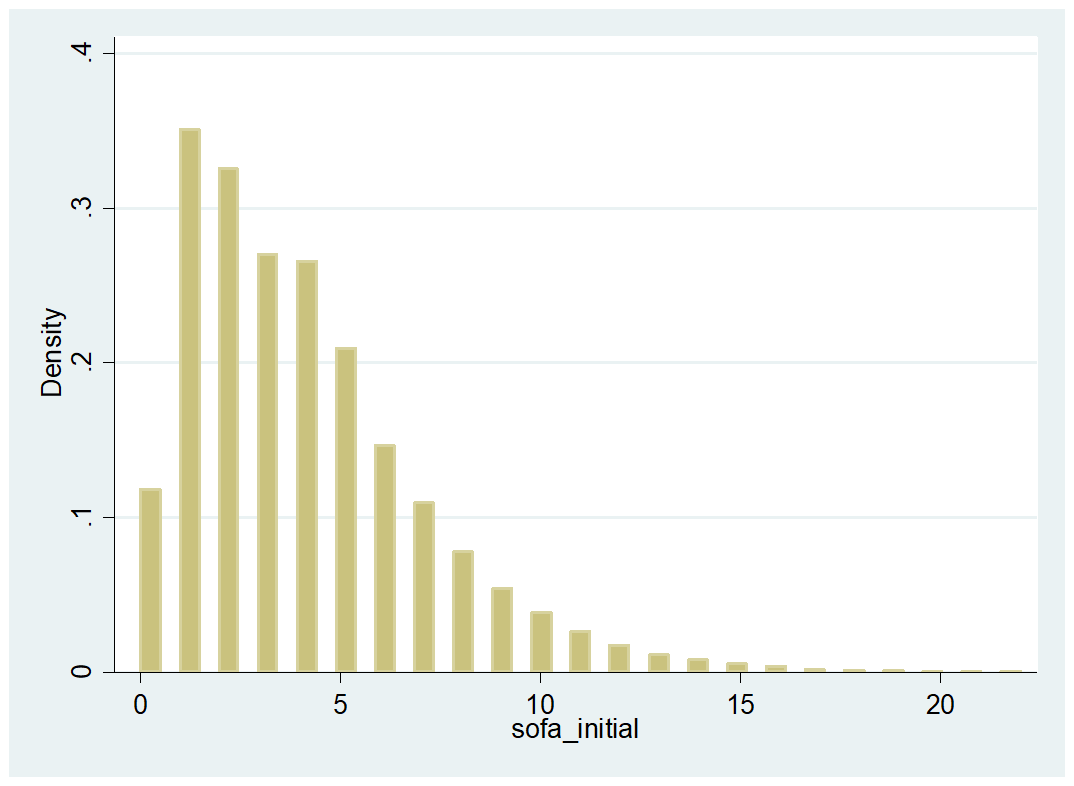

Supplement: Supplementary file 1 — Additional file 1. The distribution of SOFA score. [file 12872_2021_2039_MOESM1_ESM.tif]
